# A Superhydrophobic Gel Fracturing Fluid with Enhanced Structural Stability and Low Reservoir Damage

**DOI:** 10.3390/gels11100772

**Published:** 2025-09-25

**Authors:** Qi Feng, Quande Wang, Naixing Wang, Guancheng Jiang, Jinsheng Sun, Jun Yang, Tengfei Dong, Leding Wang

**Affiliations:** 1 College of Petroleum Engineering, China University of Petroleum (Beijing), 18 Fuxue Road, Beijing 102249, China; 18563073299@163.com (Q.F.); 15664628289@163.com (Q.W.); 18801281480@163.com (J.Y.); dtf010019@cup.edu.cn (T.D.); 2 Downhole Technical Service Branch, CNPC Bohai Drilling Engineering Co., Ltd., Tianjin 300457, China; bhdc@cnpc.com.cn; 3 CNPC Engineering Technology R&D Company Limited, Beijing 102206, China; sunjsdri@cnpc.com.cn; 4 CNPC Zhejiang Oilfield Branch, Hangzhou 310023, China; gxg0822@126.com

**Keywords:** fracturing fluid, reservoir, superhydrophobic, mechanism

## Abstract

Conventional fracturing fluids, while essential for large-volume stimulation of unconventional reservoirs, often induce significant reservoir damage through water retention and capillary trapping. To address this problem, this study developed a novel superhydrophobic nano-viscous drag reducer (SN-DR), synthesized through a multi-monomer copolymerization and silane modification strategy, which enhances structural stability and minimizes reservoir damage. The structure and thermal stability of SN-DR were characterized by FT-IR, ^1^H NMR, and TGA. Rheological evaluations demonstrated that the gel fracturing fluid exhibits a highly stable three-dimensional network structure, with a G′ maintained at approximately 3000 Pa and excellent shear recovery under cyclic stress. Performance tests showed that a 0.15% SN-DR achieved a drag reduction rate of 78.1% at 40 L/min, reduced oil–water interfacial tension to 0.91 mN·m^−1^, and yielded a water contact angle of 152.07°, confirming strong hydrophobicity. Core flooding tests revealed a flowback rate exceeding 50% and an average permeability recovery of 86%. SEM and EDS indicated that the gel formed nanoscale, tightly packed papillary structures on core surfaces, enhancing roughness and reducing water intrusion. The study demonstrates that gel fracturing fluid enhances structural stability, alters wettability, and mitigates water-blocking damage. These findings offer a new strategy for designing high-performance fracturing fluids with integrated drag reduction and reservoir protection properties, providing significant theoretical insights for improving hydraulic fracturing efficiency.

## 1. Introduction

To meet the demand for large-volume fracturing of unconventional oil and gas reservoirs, such as deep reservoirs, plastic reservoirs (rock reservoirs that undergo plastic deformation under pressure and temperature changes), and fracture-developed reservoirs, the variable viscosity fracturing fluid system has come into being [1,2]. Variable viscosity fracturing fluid allows for viscosity adjustments of the system by adjusting the content of drag reducers or introducing crosslinking agents. It has functions such as transmitting pressure, carrying proppants, and reducing formation temperature [3,4]. During the fracturing process, it mainly exhibits excellent sand carrying and drag reduction performance [5]. However, during the process of variable viscosity and smooth water fracturing, various types of reservoir damage can be caused, such as liquid phase trap damage, sensitivity damage, solid-phase invasion, and other reservoir damage [6,7]. These damages can reduce reservoir permeability in mild cases and significantly impact oil and gas production in severe cases [8].

The reservoir protection is implemented during the fracturing process to minimize reservoir damage [9]. Domestic and foreign scholars are tirelessly working on the mechanism of reservoir damage caused by fracturing fluids and the development of fracturing fluids with reservoir protection properties. Xu et al. [10] used the Danniudi gas field reservoir as an example to explore the damage mechanism of fracturing fluid in tight sandstone reservoirs. The results show that the poorer the physical properties and stronger the heterogeneity of the reservoir, the worse the backflow effect, and the more serious the damage to the reservoir. Wang et al. [11] investigated the effect of fracturing fluid on the microstructure of shale reservoirs under static adsorption, which has certain guiding significance for determining the fracturing fluid formulation parameters for reservoir protection. Zhou et al. [12] prepared a nanoparticle-modified clean fracturing fluid, which has an extremely low damage rate to the core and can significantly increase the crude oil production of low-permeability reservoirs. Wang et al. [13] developed a new type of nano multifunctional fracturing fluid, which can quickly break the gel within 20 min with extremely low residue content and has good reservoir protection effect. And the application effect feedback in Changqing Oilfield is good. Although many scholars have conducted research on reservoir protection in the field of fracturing fluids, the research on damage mechanisms and reservoir protection methods is not in-depth enough.

In this paper, a SN-DR was synthesized with reservoir protection properties to address the above problems. Firstly, the molecular structure and thermal stability of SN-DR were characterized by FT-IR, ^1^H NMR, and TGA. Secondly, the mechanism of SN-DR in reservoirs was investigated through rheological properties, thixotropic property, surface interfacial tension, capillary, surface wettability, natural imbibition, and SEM. Finally, the reservoir protection performance of SN-DR was explored through flowback rate experiment and core damage evaluation systems. Unlike conventional fracturing fluid modifiers, the proposed SN-DR combines superhydrophobic nanoparticle grafting with tailored functional polymer chains, achieving a dual functionality of drag reduction and reservoir protection. This innovative approach offers a new strategy for mitigating formation damage in hydraulic fracturing operations. This study aims to reveal the mechanism of SN-DR in reservoirs and elucidate its impact during fracturing processes. Furthermore, our findings contribute valuable insights for developing reservoir-protective fracturing fluids.

## 2. Results and Discussion

### 2.1. Characterization of SN-DR

#### 2.1.1. Fourier Transform Infrared Spectroscopy Analysis

**Figure 1:**
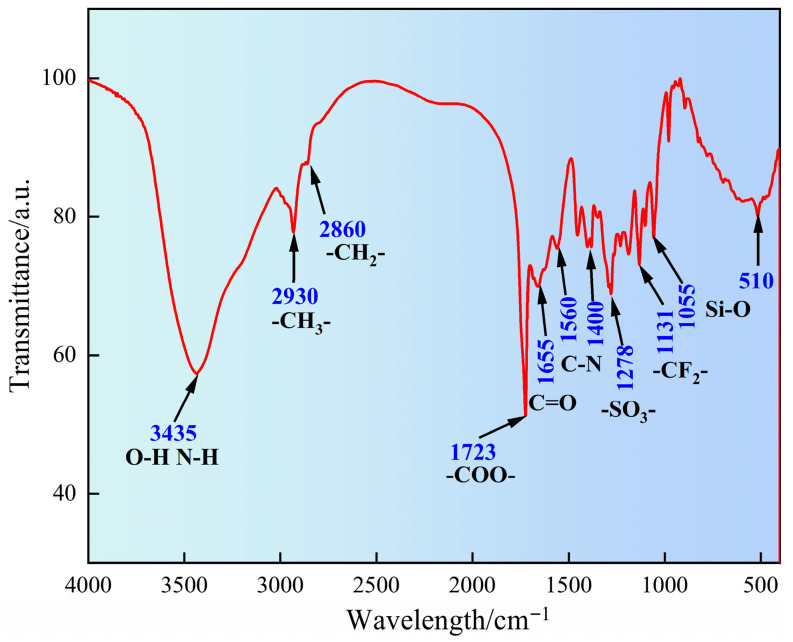
Analysis of molecular characteristic functional groups of SN-DR by Fourier transform infrared spectroscopy.

The molecular characteristic functional groups of SN-DR were analyzed by FT-IR, as shown in Figure 1. It can be seen from Figure 1 that the broad peak at 3435 cm^−1^ corresponds to O–H and N–H stretching from acrylic acid, acrylamide, and 2-acrylamido-2-methylpropanesulfonic acid. The peaks at 2930 cm^−1^ and 2860 cm^−1^ are attributed to C–H stretches from alkyl chains in all monomers, particularly the quaternary ammonium species. The strong carbonyl stretching vibration at 1723 cm^−1^ originates from the ester/carboxyl group of AA. The amide I and II bands at 1655 cm^−1^ and 1560 cm^−1^ confirm the presence of AM and AMPS. The S=O stretching vibrations at 1278 cm^−1^ and 1131 cm^−1^ are characteristic of the sulfonic acid group in AMPS, and absorptions near 1055 cm^−1^ and 510 cm^−1^ indicate siloxane-related bonds (Si–O–C/Si–O–Si), supporting the successful synthesis of the copolymer with all intended functionalities [14].

#### 2.1.2. ^1^H Nuclear Magnetic Resonance Spectroscopy Analysis

**Figure 2:**
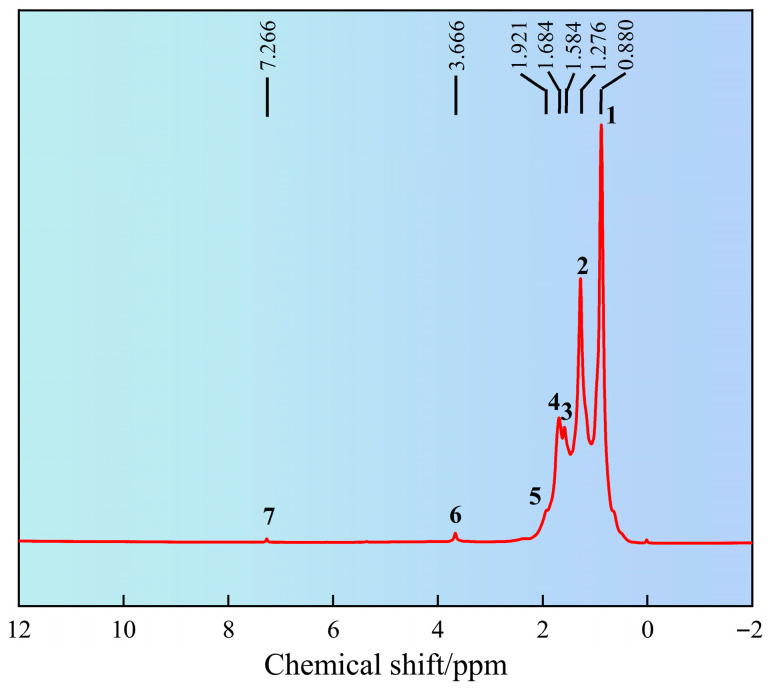
Analysis of molecular structure of SN-DR by ^1^H nuclear magnetic resonance spectroscopy.

The chemical shift of hydrogen atoms in the synthesized product SN-DR was analyzed by ^1^H NMR, as shown in Figure 2. It can be seen from Figure 2 that the chemical shift at peak position 1 was 0.880 ppm, corresponding to CH_3_. The chemical shift at peak position 2 was 1.276 ppm, corresponding to CH_2_. The chemical shift at peak position 3 was 1.584 ppm, corresponding to CH. The chemical shift at peak position 4 was 1.684 ppm, corresponding to CH. The chemical shift at peak position 5 was 1.921 ppm, corresponding to CH_3_ near SiO_2_. The chemical shift at peak position 6 was 3.666 ppm, corresponding to CH_3_ near the quaternary ammonium salt. The chemical shift at peak position 7 was 7.266 ppm, corresponding to N-H [15]. Therefore, through ^1^H NMR and FT-IR, it can be concluded that the synthesized product SN-DR had a molecular structure consistent with the expected design, indicating successful synthesis.

#### 2.1.3. Thermogravimetric Analysis

**Figure 3:**
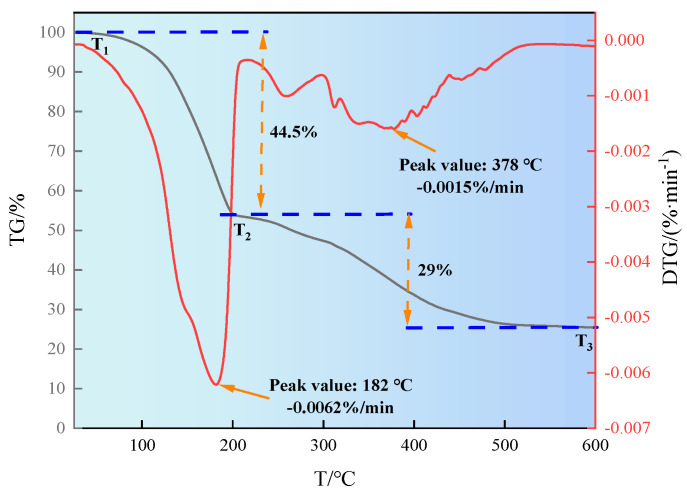
Study on thermal stability of SN-DR by thermogravimetric analysis.

The thermal stability of the synthesized product SN-DR was analyzed, and the experimental results are shown in Figure 3. It can be seen from Figure 3 that the thermogravimetric curve of SN-DR is divided into two stages. In the first stage, the quality loss from T_1_ to T_2_ was 44.5%. The maximum thermal decomposition rate was 0.0062 %/min at 182 °C. The weight loss in this stage is mainly attributed to the evaporation of adsorbed water on the product surface and the breakage of molecular branches. In the second stage, the quality loss from T_2_ to T_3_ was 29%. The maximum thermal decomposition rate was 0.0015 %/min at 378 °C. The quality loss in this stage is mainly attributed to the thermal decomposition caused by the breakage of some molecular main chains [16]. The results indicated that SN-DR had good thermal stability.

#### 2.1.4. Microscopic Morphology

**Figure 4:**
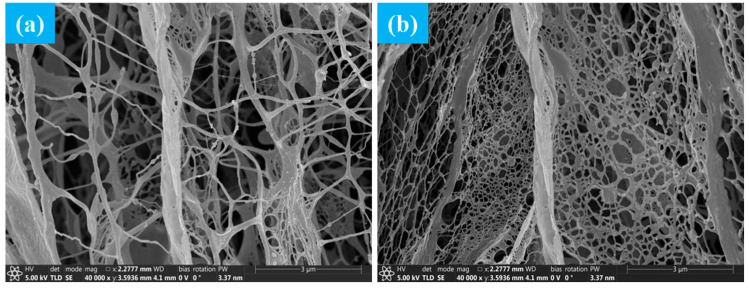
Microstructure of gel fracturing fluid. (**a**) is the microstructure of ordinary polyacrylamide fracturing fluid. (**b**) is the microstructure of superhydrophobic fracturing fluid.

The differences in microstructure between ordinary polyacrylamide and superhydrophobic gel fracturing fluid have been studied. The gelatinized gel skeleton was characterized by SEM. Figure 4a,b show the microstructure of ordinary polyacrylamide fracturing fluid and superhydrophobic fracturing fluid, respectively.

It can be seen from Figure 4a that the three-dimensional network structure formed by ordinary polyacrylamide fracturing fluid was relatively loose, consisting of a random entangled network of fibrous polymer segments. This structure had the following characteristics: (1) Sparse network nodes and lack of a stable crosslinked skeleton. (2) Uneven distribution of pores, with large open pores in some areas. (3) The chain segments were slender and soft, the network rigidity was insufficient, and the ability to resist deformation was limited. (4) The arrangement of local segments was disordered, resulting in low structural density. In addition, this gel structure had weak response to external disturbances (such as shear, high pressure), and limited stability and viscoelastic properties [17]. However, it can be seen from Figure 4b that the three-dimensional crosslinked network structure of the superhydrophobic gel fracturing fluid was relatively dense. The network structure was significantly thickened, the distribution of chain segments was denser, and the overall skeleton connectivity was enhanced. The pore scale was significantly reduced and evenly distributed, and the structural continuity and rigidity were improved. This was mainly attributed to spherical nanoparticles with a diameter of about 100–200 nm acting as physical crosslinking points, entangling with chain segments to form stable anchoring structures. Meanwhile, the chain bridging effect induced by particles enhanced the orderliness of local arrangement, significantly improving the stability and shear recovery ability of the network structure.

### 2.2. Rheological Properties of Gel Fracturing Fluid

**Figure 5:**
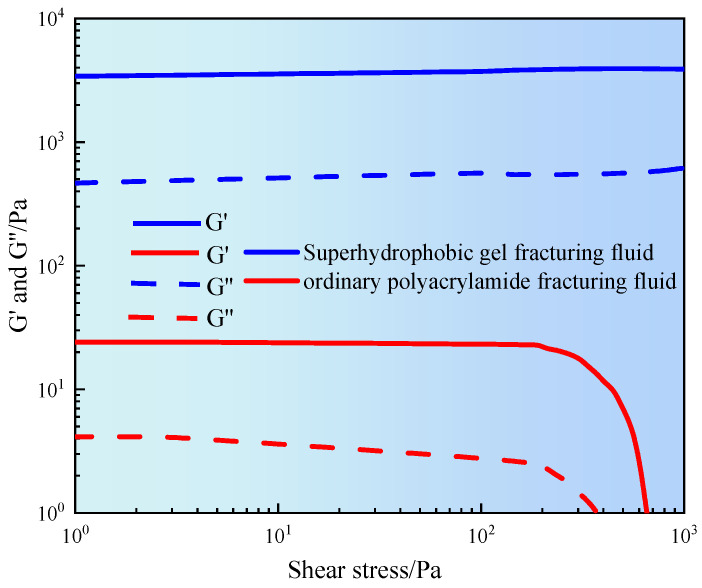
Stress sweep curve of gel fracturing fluid.

The structural stability and viscoelastic evolution of fracturing fluid under different shear strengths have been studied. Figure 5 shows the relationship between the storage modulus (G′) and loss modulus (G″) of fracturing fluid and shear stress.

It can be seen from Figure 5 that the G′ and G″ of ordinary polyacrylamide fracturing fluid remained stable in the low shear stress region (<100 Pa). When in the linear viscoelastic range, the physical entanglement or weak crosslinking structure between polymer segments had not been disturbed. As the shear stress increased, especially between 100–1000 Pa, both moduli gradually decreased. When entering the nonlinear response zone, the polymer network structure gradually disintegrated and its ability to resist deformation weakened. The results indicated that ordinary fracturing fluid had poor structural stability and was prone to shear damage in high stress environments [18]. However, superhydrophobic gel fracturing fluid exhibited high modulus levels throughout the entire range of shear stress. The G′ of superhydrophobic gel fracturing fluid was maintained at about 3000 Pa, and the G″ was stable around 700 Pa. In addition, the amplitude of their changes with shear stress was relatively small, and the overall curve tended to be stable. The results indicated that the superhydrophobic gel fracturing fluid had a highly stable network structure, good shear resistance, and deformation recovery ability. This was mainly attributed to the introduction of hydrophobic nanoparticles, which formed crosslinked nodes between polymer chains. By utilizing multiple mechanisms such as hydrophobic association and particle bridging, a stable three-dimensional spatial framework was constructed, significantly enhancing the elastic energy storage capacity and energy dissipation performance of the gel fracturing fluid [19].

The stability and viscoelastic characteristics of the fracturing fluid network structure are key indicators for evaluating its sand carrying performance and flow resistance. Therefore, it is necessary to study the variation of storage modulus (G′) and loss modulus (G″) of fracturing fluid at frequencies of 0.1–100 Hz.

**Figure 6:**
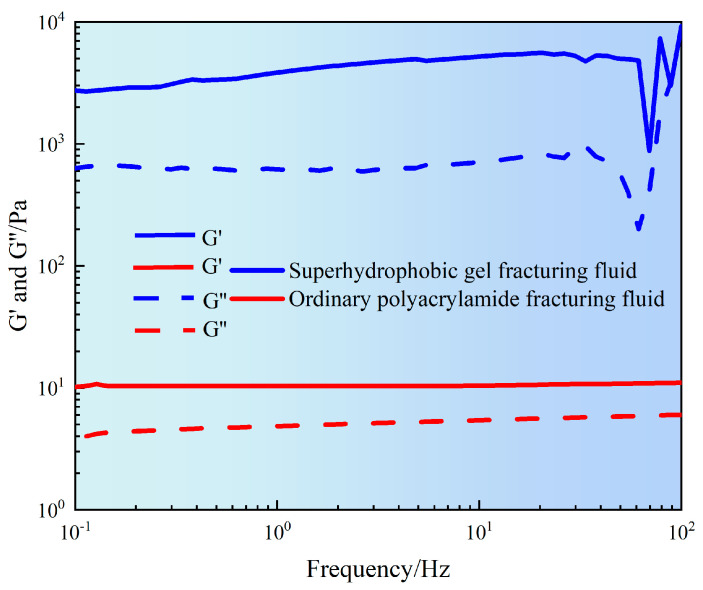
Frequency sweep curve of gel fracturing fluid.

It can be seen from Figure 6 that the G′ of ordinary polyacrylamide fracturing fluid was basically maintained within the range of 15–30 Pa, while the G″ was within the range of 5–10 Pa. Both moduli slightly increased with increasing frequency, indicating that the system had a loose structure and low crosslink density. This may mainly rely on the entanglement of chain segments to form a weakly elastic network structure. Although slightly enhanced in the high-frequency region, the overall performance was a low modulus, low-energy system that was prone to instability under high shear conditions. However, superhydrophobic gel fracturing fluid exhibited high modulus throughout the entire frequency range. Among them, G′ slowly increased from 3000 Pa to 4000 Pa, and G″ increased from 700 Pa to 900 Pa. The modulus slowly increased with the increase of frequency, indicating that the superhydrophobic fracturing fluid formed a highly stable three-dimensional network structure. This may be due to the three-dimensional network structure formed by hydrophobic interactions between particles, particle adsorption, and physical bridging. In addition, the G′ of superhydrophobic fracturing fluid was significantly higher than G′ (there is an order of magnitude difference between G′ and G″ modulus). This indicated that superhydrophobic gel fracturing fluid was an elastic dominant fluid, which would help achieve excellent sand suspension ability. However, the G′ of ordinary polyacrylamide fracturing fluid was close to G″, indicating that its structural strength was weak and could not form a stable support network. The results indicated that superhydrophobic gel fracturing fluid not only had a high modulus level, but also maintained good structural stability under dynamic loads, providing theoretical basis and experimental support for the application of subsequent fracturing fluid in high shear and high-frequency environments [20].

### 2.3. Thixotropic Properties of Gel Fracturing Fluid

**Figure 7:**
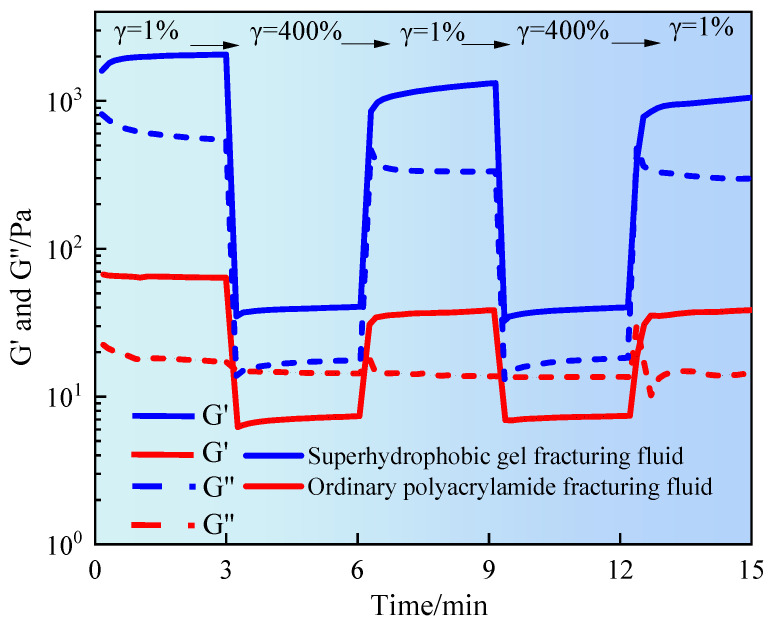
Thixotropic test of gel fracturing fluid.

This experiment simulates the periodic shear disturbances experienced by fracturing fluid in dynamic load environments such as pump start-up, throttling impact, and wellbore backpressure. The structural stability and self-healing ability were evaluated. Figure 7 shows the temporal variation of G′ and G″ of fracturing fluid under alternating shear conditions.

It can be seen from Figure 7 that the superhydrophobic gel fracturing fluid exhibited significant elastic characteristics in the initial stage. G′ was stable at about 1200 Pa, much higher than G″, indicating that the superhydrophobic gel fracturing fluid was in a highly elastic state. At the high strain stage (γ = 400%), both moduli rapidly decreased to 30–50 Pa, indicating that the three-dimensional network structure was sheared and destroyed. However, in the low strain stage, the modulus could quickly recover to its initial level and exhibited recovery in multiple consecutive shear cycles. This was mainly due to the multiple physical interaction mechanisms within the system, including hydrophobic group association, polymer segment entanglement and nanoparticle bridging, which effectively supported the reconstruction ability of the gel network [21]. However, the initial modulus of ordinary polyacrylamide fracturing fluid was significantly lower, with G′ and G″ being 25 Pa and 15 Pa, respectively. This indicated that the structure of this system primarily relied on weak entanglement and hydrogen bonding interactions. Each high-strain shear event caused a sharp decline in modulus, with limited recovery during subsequent low-strain stages. This exhibited typical irreversible structural damage characteristics. The sustained decline in modulus indicated that the system lacks effective crosslinking points or reconfigurable structures. Once the structure was damaged, it was difficult to restore it to its original state. This directly affected its sand-carrying capacity and its adaptability under complex reservoir conditions or multiple pumping cycles. Superhydrophobic gel fracturing fluid exhibited excellent viscoelasticity and structural self-healing properties under periodic shear disturbances. It has been confirmed that superhydrophobic nanoparticles played an important role in enhancing the dynamic stability and network reconstruction ability of fracturing fluids. At the same time, the high modulus and high recovery performance of superhydrophobic fracturing fluid had better stability and engineering application prospects in complex formation fracturing operations [22].

### 2.4. Drag Reduction Performance

**Figure 8:**
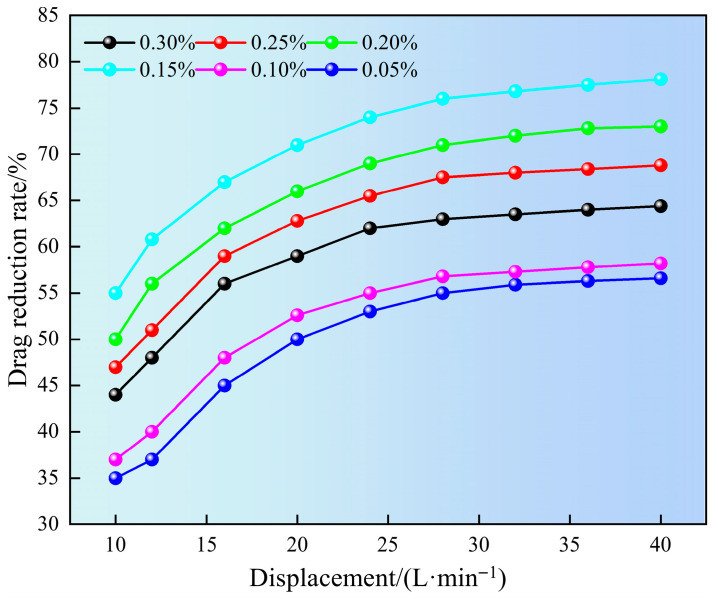
Impact of displacement on drag reduction rate.

Figure 8 shows the effect of superhydrophobic gel fracturing fluid on drag reduction rate at different displacements. It can be seen from Figure 8 that as the displacement increased, the drag reduction rate also gradually increased. This may be attributed to the increase in displacement leading to an increase in flow velocity within the pipe column. As the flow rate increased, the SN-DR molecules expanded and interacted with vortices, suppressing turbulent pulsations and significantly increasing the drag reduction rate. However, when the flow rate exceeded the critical value, molecular structure of gel might undergo chain breakage under high shear, which affected the drag reduction rate. In addition, the drag reduction rate of 0.15% SN-DR reached 78.1% at a displacement of 40 L/min. This may be attributed to two aspects: on the one hand, appropriate liquid viscosity could suppress turbulent fluctuations and had a more significant drag reduction effect. However, high liquid viscosity might suppress turbulence and reduce drag reduction potential. On the other hand, SN-DR would form a dynamic adsorption layer near the pipe wall, reducing direct contact between liquid and solid surfaces and significantly reducing wall friction resistance [23].

### 2.5. Surface Tension and Oil–Water Interface Tension

**Figure 9:**
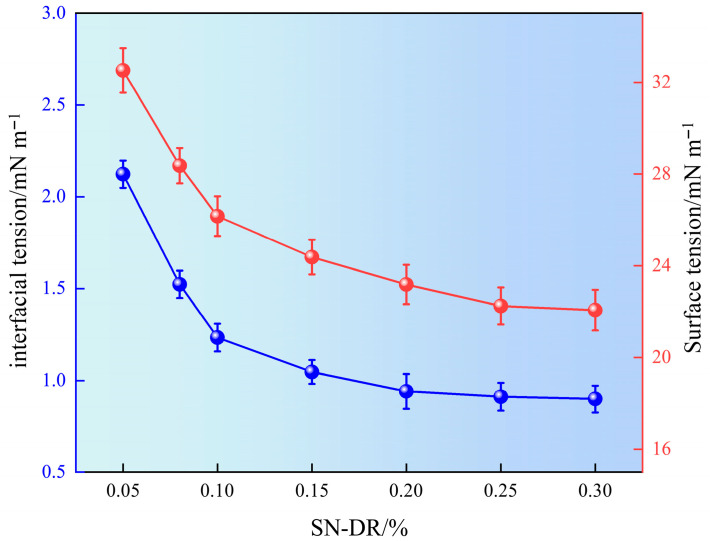
Surface interfacial tension of drag reducing agent solution.

After fracturing, it is necessary to promptly return the fracturing fluid to the surface. Therefore, fracturing fluids are required to have relatively low surface tension, which can effectively reduce the retention time in the reservoir and achieve the goal of reducing reservoir damage. The superhydrophobic gel fracturing fluid was prepared with different concentrations of drag reducer. The surface tension and interfacial tension of each system were measured, and the experimental results are shown in Figure 9.

It can be seen from Figure 9 that as the concentration of SN-DR increased, the surface tension decreased from 31.51 mN m^−1^ to 22.07 mN m^−1^, which was 30% lower. The interfacial tension of SN-DR decreased from 2.45 mN m^−1^ to 0.91 mN m^−1^, which was 62.8% lower. Meanwhile, when the amount of SN-DR was 0.2%, the changes of surface and interfacial tension tended to level off. The results showed that SN-DR could reduce surface interfacial tension. The main reason was the presence of long-chain carbon and sulfonic acid groups in SN-DR. Long chain carbon groups had hydrophobic properties, while sulfonic acid groups had hydrophilic properties. In addition, they could interact with water molecules to form strong hydrogen bonds and further reduce surface tension. Therefore, the synergistic effect of long-chain carbon and sulfonic acid groups in SN-DR molecules reduced their surface energy, which helped to improve the reflux rate of smooth hydraulic fracturing fluid and achieve reservoir protection [24].

### 2.6. Capillary Experiment

**Figure 10:**
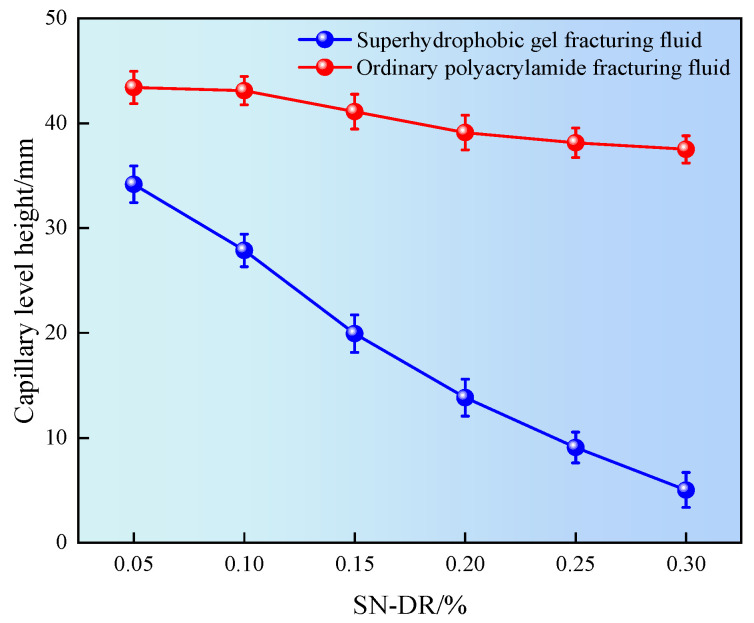
Liquid level height in capillary tube.

Micro nano pore throats are developed in shale reservoirs, which are prone to natural seepage and absorption when exposed to water, causing reservoir damage to the pore throats. In addition, hydrophobic groups (such as long-chain alkyl groups) have lower surface energy compared to hydrophilic groups. When these hydrophobic groups exist on the surface of capillaries, they will reduce the total surface energy of the solid. In capillaries that rely on the balance of adhesion and cohesion, surfaces with hydrophobic groups will exhibit reduced capillary action. This means that compared to hydrophilic capillaries, the liquid rise in hydrophobic capillaries is smaller, because weak adhesion cannot overcome the cohesive force that keeps the liquid inside [25]. Therefore, the influence of superhydrophobic gel fracturing fluid on the capillary liquid level height was investigated by simulating the core pore throat through capillary tubes, as shown in Figure 10.

It can be seen from Figure 10 that the capillary liquid surface height after being immersed in the superhydrophobic gel fracturing fluid was significantly lower than that the ordinary polyacrylamide fracturing fluid. Meanwhile, when the dosage of SN-DR was 0.05%, the capillary liquid level height was 35 mm. When the dosage was 0.3%, the capillary liquid level height was 5 mm. The results showed that the height of the capillary liquid level decreased with the increase of the concentration of drag reducer. The main reason was that the hydrophobic groups in the SN-DR were adsorbed on the inner wall of the capillary, causing the inner wall of the capillary to change from hydrophilic to hydrophobic, which could significantly reduce the liquid level height in the capillary [26].

### 2.7. Surface Wettability

**Figure 11:**
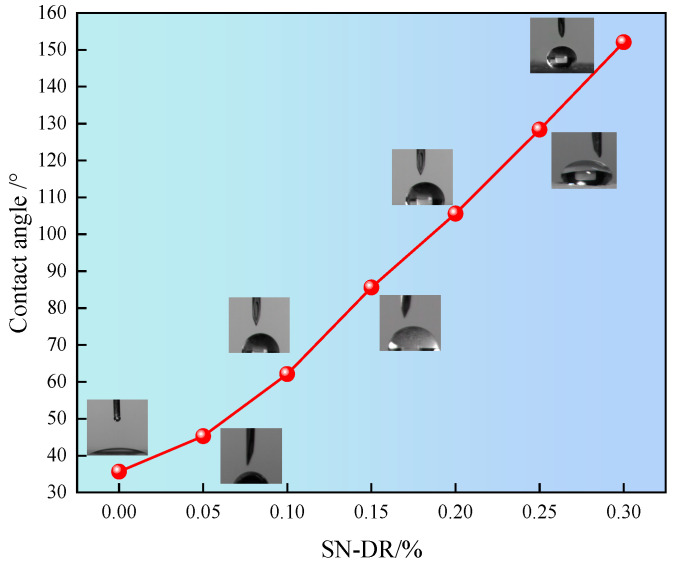
Influence of SN-DR on surface wettability of core.

The effect of fracturing fluid breaking process on the wettability of rock core surface is complex and important. Generally speaking, when the contact angle is less than 90°, the liquid spreads well on the surface, indicating hydrophilicity. When the contact angle is greater than 90°, the liquid forms water droplets on the surface, indicating hydrophobicity. The effect of superhydrophobic gel fracturing fluid on the wettability of the core surface was tested, as shown in Figure 11.

It can be seen from Figure 11 that as the concentration of SN-DR increased, the contact angle of the water phase became larger and larger. When the dosage of SN-DR was 0.3%, the contact angle of the water phase was 152.07°. The results indicated that the SN-DR could improve the wettability of the reservoir. This was mainly attributed to the fact that SN-DR molecules could be adsorbed on the surface of core, achieving wetting flipping. The wettability of the reservoir surface had changed from hydrophilic to hydrophobic, which could effectively prevent the penetration of water molecules and water lock damage [27].

### 2.8. Natural Imbibition

**Figure 12:**
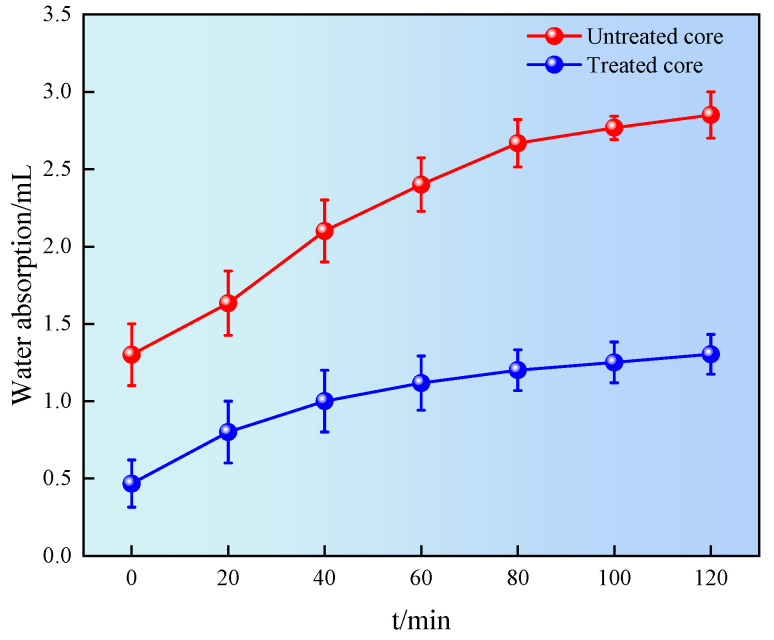
Relationship between natural imbibition and time.

A spontaneous imbibition test can be conducted to evaluate the effect of the wettability on fluid imbibition into the shale. The shale was immersed in a liquid, and the amount of liquid absorbed was recorded on a balance as a function of time, as shown in Figure 12.

It can be seen from Figure 12 that the water absorption of shale first increased and then tended to stabilize. The imbibition volume of untreated shale was 2.85 mL at 120 min. The imbibition volume of treated shale was 1.21 mL at 120 min, a decrease of 57.5% compared to the untreated shale. The results showed that the wettability of shale surface treated with superhydrophobic gel fracturing fluid was changed from hydrophilicity to hydrophobicity, reducing the possibility of water lock damage in shale pores and throats, and achieving the effect of reservoir protection [28].

### 2.9. Flowback Rate Experiment

**Figure 13:**
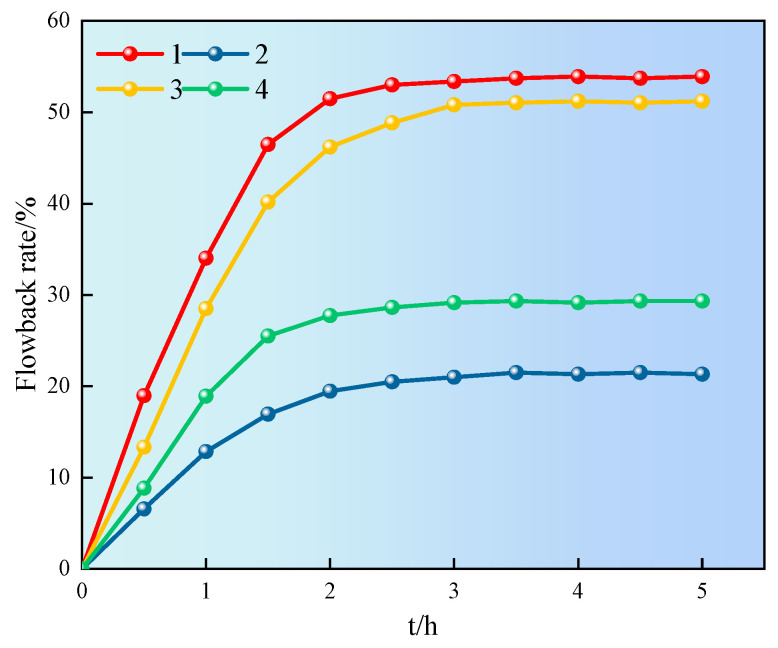
Influence of different solutions on flowback rate.

During the fracturing process, the flowback rate of the hydraulic fracturing fluid is enormous. In order to achieve the goals of energy conservation and environmental protection, the flowback liquid can be recycled multiple times. The effective components in the flowback liquid can be reused to the maximum extent, which can appropriately reduce the amount of additives and waste of water resources. Therefore, the effect of superhydrophobic gel fracturing fluid at 60 °C and 4.5 MPa on the core flowback was investigated, as shown in Figure 13.

It can be seen from Figure 13 that the flowback rate of the core first increased and then tended to stabilize. The flowback rate of cores 1 and 3 was significantly higher than that of cores 2 and 4, reaching over 50%. The difference in the flowback rate between cores 1 and 3, 2 and 4 was mainly due to the different cross-sections of the core, and the distribution of pore throats and fractures had a certain impact on flowback. The results showed that the superhydrophobic gel fracturing fluid could alter the flowback rate of core. The SN-DR was adsorbed on the surface of core, reducing surface free energy and capillary force reversal, which could effectively reduce water lock damage [29].

### 2.10. Evaluation of Core Damage Performance

The recovery value of core permeability plays an important guiding role in on-site fracturing, directly affecting the design, implementation, and later production prediction and optimization of fracturing operations. The reservoir damage should be minimized to the greatest extent possible during the fracturing process. However, there are significant differences in the properties of fracturing fluids themselves, which can still cause varying degrees of damage to the reservoir. Therefore, the damage performance of superhydrophobic gel fracturing fluid on core was evaluated, and the experimental results are shown in Table 1.

It can be seen from Table 1 that the average permeability recovery value of the superhydrophobic gel fracturing fluid on the core was 86.0%. The results showed that the superhydrophobic gel fracturing fluid could exhibit good reservoir protection effect in fracturing operations. Moreover, the SN-DR was adsorbed in the pore throats on the core surface, preventing the retention and intrusion of infiltrating water [30].

### 2.11. Scanning Electron Microscope

**Figure 14:**
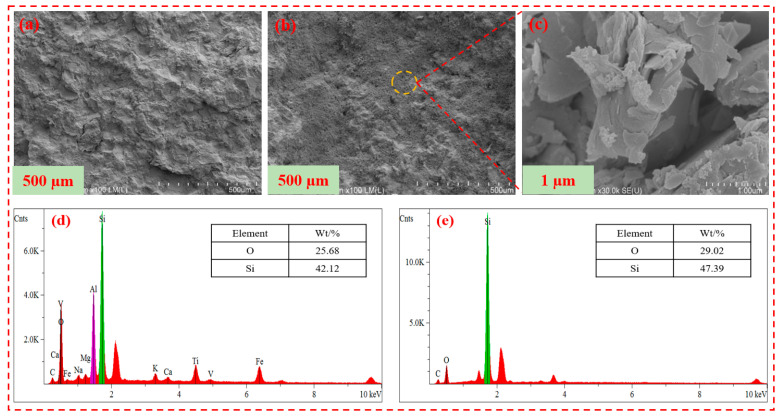
Surface analysis of core treated with fracturing fluid. (**a**) is the microstructure of the core surface treated with ordinary polyacrylamide fracturing fluid. (**b**,**c**) are the microscopic morphology of the core surface treated with superhydrophobic fracturing fluid. (**d**) is the EDS of the core surface treated with ordinary polyacrylamide fracturing fluid. (**e**) is the EDS of the core surface treated with superhydrophobic fracturing fluid.

The surface analysis of the core treated with ordinary polyacrylamide fracturing fluid and superhydrophobic gel fracturing fluid is shown in Figure 14. It can be seen from Figure 14a that the surface of the core treated with ordinary polyacrylamide was uneven. It can be seen from Figure 14b that the surface of the core treated with superhydrophobic gel fracturing fluid had formed a densely arranged papillary structure, which increased the surface roughness of the core and hindered the invasion of foreign liquid phases. It can be seen from Figure 14c that the SN-DR was adsorbed onto the surface of the core, and its nano-micron size did not cause blockage of the core pore throats [31,32].

In addition, EDS analysis was performed on the surface of the core. It can be seen from Figure 14d,e that the energy spectrum intensity of silicon and oxygen elements on the surface of the core treated with superhydrophobic gel fracturing fluid was significantly higher than that of the core surface treated with ordinary polyacrylamide fracturing fluid. Meanwhile, the surface of the core treated with superhydrophobic gel fracturing fluid had more silicon and oxygen elements. The results showed that the SN-DR could adsorb well on the surface of core, hindering the invasion of liquid phase [33].

### 2.12. Mechanism Analysis

**Figure 15:**
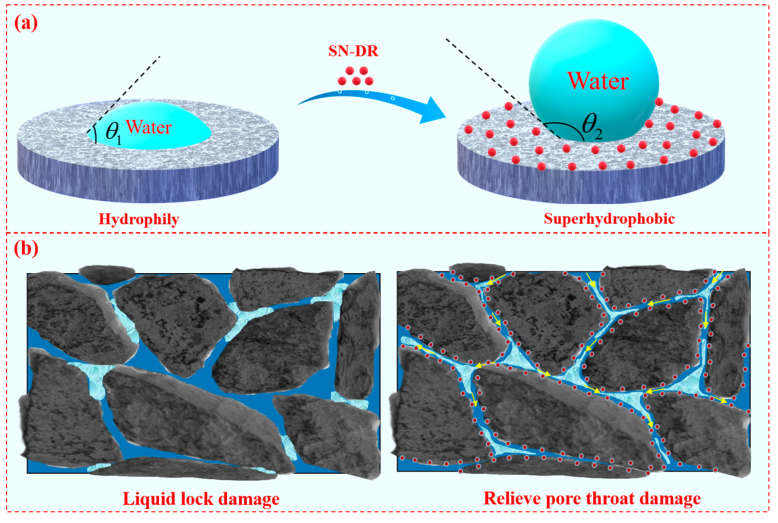
Mechanism of SN-DR in reservoirs. (**a**) is the wettability of the water phase on the surface of the rock core before and after treatment with superhydrophobic fracturing fluid. (**b**) is a demonstration diagram of liquid lock damage before and after treatment with superhydrophobic fracturing fluid.

The SN-DR was adsorbed on the surface of reservoir rock by physicochemical means, which made the wettability of the rock surface change and reduced the capillary force and waterproof lock damage [34,35]. At the same time, the friction of formation fluid flow was reduced under the influence of SN-DR, which increased the relative permeability of formation fluid. It can be seen from Figure 15a that the contact angle of water on the core was small, and the core surface had strong hydrophilicity. After the core was soaked by the superhydrophobic gel fracturing fluid, not only the surface energy was reduced, but also the SN-DR was adsorbed on the core surface under the effect of electrostatic and hydrogen bonding and so on. Meanwhile, the surface of core was transformed from hydrophilic to hydrophobic, exhibiting a large water-phase contact angle [36,37].

It can be seen from Figure 15b that the core was not treated by superhydrophobic gel fracturing fluid, and when a small amount of liquid passed through, it would be adhered to the surface of the pore throat. Due to the strong friction between the liquid and the surface of the pore throat, the liquid was gathered and retained in the pore throat, resulting in the blockage of the pore throat channel and the reduction of permeability [38]. When the core was treated with superhydrophobic gel fracturing fluid, firstly, SN-DR would enter the rock pore throats under the influence of external pressure, temperature, and capillary forces. Secondly, SEM confirmed that SN-DR could adsorb on the surface of rock cores. It was confirmed through surface wettability that the core surface achieved wetting flipping. Through core displacement experiments, it was confirmed that the superhydrophobic gel fracturing fluid polluted core had good permeability recovery values, showing good fluidity of oil and gas in the core pore throats. Therefore, it can be confirmed that the SN-DR could reduce the frictional force of oil and gas flow in the core pore throat and improve the efficiency of oil and gas flow. It played the role of shale reservoir protection and realized the purpose of improving the recovery rate [39,40,41].

## 3. Conclusions

In this study, a novel superhydrophobic nano-viscous drag reducer (SN-DR) was successfully synthesized and its performance as a key component in gel fracturing fluid was comprehensively evaluated. The main conclusions are as follows:

(1) Successful Synthesis and Robust Structure: SN-DR was successfully synthesized via multi-monomer copolymerization and silane modification, as confirmed by FT-IR and ^1^H NMR analyses. The material demonstrated good thermal stability.
(2) Enhanced Rheological Properties: The gel fracturing fluid formulated with SN-DR exhibited a highly stable three-dimensional network structure, characterized by a high storage modulus (3000 Pa) that was significantly greater than that of conventional polyacrylamide fluid. It demonstrated exceptional shear recovery and thixotropic behavior under cyclic stress, indicating superior structural stability and self-healing capability.
(3) Excellent Drag Reduction and Interfacial Activity: A low concentration of 0.15% SN-DR achieved a high drag reduction rate of 78.1% at 40 L/min. Furthermore, it effectively reduced the surface tension (to 22.07 mN·m^−1^) and oil–water interfacial tension (to 0.91 mN·m^−1^), facilitating flowback and reducing capillary pressure.
(4) Effective Wettability Alteration and Water Block Mitigation: SN-DR significantly altered the wettability of the core surface to superhydrophobic, achieving a water contact angle of 152.07°. This led to a 57.5% reduction in spontaneous water imbibition and a marked decrease in capillary rise height, confirming its efficacy in mitigating water-blocking damage.
(5) Superior Reservoir Protection Performance: Core flooding tests demonstrated outstanding reservoir protection, with an average permeability recovery value of 86.0% and a flowback rate exceeding 50%. Microscopic analysis (SEM/EDS) revealed that SN-DR formed nanoscale, tightly-packed papillary structures on the core surface, increasing roughness and forming a protective barrier that hinders water intrusion without plugging pore throats.

Future studies will employ statistical design-of-experiment approaches, such as Response Surface Methodology (RSM), to optimize the synthesis parameters for scaled-up production and enhanced performance in large-scale field applications.

In summary, the SN-DR-based gel fracturing fluid developed in this work integrates the functions of efficient drag reduction and robust reservoir protection into a single system. It presents a promising strategy for minimizing formation damage and enhancing the efficiency of hydraulic fracturing in unconventional reservoirs.

## 4. Materials and Methods

### 4.1. Materials

Ethanol (99%), nano-silica (99%), paraffin (EP), sodium carbonate (CP), ammonium persulfate (99.9%), silane coupling agent (KH-550, AR), N-octyltriethoxysilane (97%), and silane coupling agent (KH-570, AR) were purchased from Shanghai Anakee Chemical Co., Ltd., Shanghai, China. Emulsifier TX-3 (CP), Emulsifier TX-10 (CP), EDTA-2Na (AR), Span-80 (AR), V50 initiator (AR), and AMPS (CP) were purchased from Bailiwick Technology Co., Ltd., Beijing, China. Acrylic acid (AR), acrylamide (99%), 2-(Methacryloyloxy)-N,N,N-trimethylammonium chloride (AR), and chloroform (99.8%) were purchased from Anhui Banghao Chemical Co., Ltd., Bengbu, China. Clean up additive (BC-ZP), general inhibitors (BC-YZJ), and gel breaker (BC-PJ) were used from Beijing Shida Bocheng Technology Co., Ltd., Beijing, China.

### 4.2. Synthesis of SN-DR

**Figure 16:**
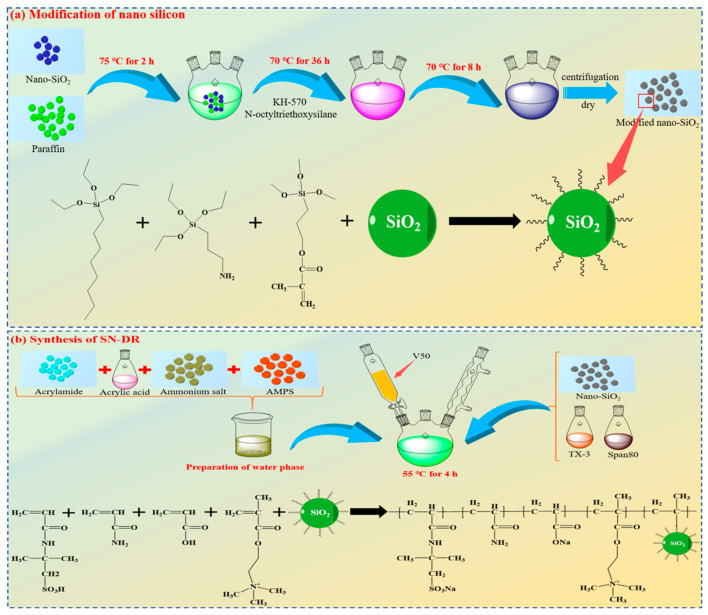
Modification of nano silicon and synthesis of SN-DR.

(1) A mass of 1 g of nano silica and 20 g of paraffin were added to a three necked flask containing 100 mL of bis (decaalkyl) dimethyl ammonium bromide solution, and stirred continuously at 75 °C for 2 h. After being cooled, the surface of the paraffin was rinsed with deionized water. The processed paraffin was added to 50 mL of ethanol, and silane coupling agent (KH-570) and N-octyltriethoxysilane were added. The reaction was carried out at 70 °C for 36 h. After being cooled, the paraffin wrapped in silica was rinsed with deionized water. The processed paraffin was added to 200 mL of chloroform solution and heated to 40 °C to dissolve the paraffin. The wax balls were collected by centrifugation and dried to constant weight at 50 °C. The dried silica and KH-570 were added to an ethanol ammonia solution and reacted at 70 °C for 8 h. After the reaction was completed, the modified silica particles were obtained by centrifugation and dried to constant weight.

(2) The modified silica particles, emulsifier TX-3, and Span-80 were added to D100 solvent oil and stirred at high speed for 10 min until evenly dispersed.

(3) The acrylic acid, acrylamide, 2-(Methacryloyloxy)-N,N,N-trimethylammonium chloride, and AMPS were added to distilled water. The pH of the mixed solution was adjusted to neutral. Then, emulsifier TX-10, and EDTA-2Na were added to the mixture and stirred for 50 min.

(4) The stirring of the oil phase flask was turned on and N_2_ was introduced. The aqueous solution was dropped into the oil phase and was completed within 30 min.

(5) The initiator V50 was slowly added and reacted at 55 °C for 4 h. At the end of the reaction, superhydrophobic nano-viscous drag reducers (SN-DR) were obtained. The reaction equation is shown in Figure 16.

(6) Following the polymerization reaction, the resulting particles were subjected to an extensive washing protocol to remove any physisorbed monomers, oligomers, or polymer chains that were not covalently attached to the silica surface. This was achieved through a series of centrifugation and redispersion cycles (typically 5–7 times) using a variety of solvents. The washing sequence employed was: deionized water, followed by ethanol/water mixture (1:1 *v*/*v*), and finally pure ethanol. Each centrifugation step was performed at 10,000 rpm for 20 min. The purified product was then dried under vacuum at 60 °C for 24 h prior to further characterization. This stringent process ensures that the characterization data (FT-IR, TGA, ^1^H NMR) presented in this work are representative of the covalently grafted polymer layer and not interfered with by physisorbed species.

### 4.3. Preparation of Superhydrophobic Gel Fracturing Fluid

Firstly, a certain amount of SN-DR, 0.3% BC-ZP, and 0.3% BC-YZJ were sequentially added to tap water and stirred at high speed for 1 h until fully dissolved to prepare superhydrophobic gel fracturing fluid [42]. Then, the superhydrophobic gel fracturing fluid was left to stand at room temperature for 24 h before being set aside.

### 4.4. Characterization Method

#### 4.4.1. Fourier Transform Infrared Spectroscopy

The molecular characteristic functional groups of SN-DR were analyzed by FT-IR using a Nicolet 6700 spectrometer (Thermo Fisher Scientific, Waltham, MA, USA). The sample was processed using potassium bromide pellet method and scanned at wavelengths ranging from 4000 cm^−1^ to 400 cm^−1^.

#### 4.4.2. ^1^H Nuclear Magnetic Resonance Spectroscopy

The molecular structure of SN-DR was analyzed by ^1^H NMR using a Bruker AV 600 M NMR spectrometer (Bruker BioSpin GmbH, Ettlingen am Rhein, Germany). The sample was dissolved in CDCl_3_ and placed in NMR tube to scan the NMR hydrogen spectrum.

#### 4.4.3. Thermogravimetric Analysis

The thermal stability of SN-DR was analyzed using a STA 449 F3 thermogravimetric analyzer (NETZSCH, Selb, Bavaria, Germany). The nitrogen flow rate was 50 mL/min. The heating rate was 10 °C/min. The sample was placed in a crucible and heated from room temperature to 600 °C. The mass loss and decomposition rate of the sample at different temperatures were recorded.

### 4.5. Geological Background of the Studied Shale

The core samples evaluated in this study were from the Longmaxi Formation in the Da’an Block of the Sichuan Basin, China. This is a representative unconventional shale reservoir that heavily relies on hydraulic fracturing for economic production. It can be seen from Table 2 and Table 3 that this formation is characterized by its complex lithology, comprising high clay content (predominantly illite and mixed-layer illite/smectite), significant quartz and carbonate minerals, and abundant organic matter. Petrophysically, it exhibits ultra-low porosity and nanodarcy-scale permeability, with well-developed natural fractures that enhance reservoir complexity but also increase susceptibility to water trapping and clay swelling. These geological attributes—low permeability, fracture networks, and reactive mineralogy—directly influence fracturing fluid behavior and underscore the necessity for tailored, low-damage fluid systems such as the one developed herein.

### 4.6. Measurement of Surface Tension and Oil–Water Interfacial Tension

According to the industry standard SY/T5370-2018 “Surface and Interface Tension Measurement Method” [43], solutions of SN-DR with different concentrations were prepared, and the surface tension of the solution was measured by using QBZY-2 fully automatic surface/interface tensiometer based on the platinum plate method at 30 °C. The interfacial tension of the solution was measured by using a rotating droplet interfacial tension meter [44].

### 4.7. Capillary Experiment

The effect of SN-DR on the wettability of shale surface was evaluated by simulating the micro pore throats inside the core through capillary experiments [45]. Firstly, the different concentrations of superhydrophobic gel fracturing fluid and ordinary polyacrylamide fracturing fluid were prepared for later use. Then, a glass capillary tube with an inner diameter of 0.1 mm was immersed in fracturing fluid for 30 min and dried. Finally, the dried capillary tube was placed in clean water, and the liquid level in the capillary tube was observed and measured.

### 4.8. Surface Wetting Performance

The different concentrations of superhydrophobic gel fracturing fluid were prepared. The cores were immersed in the solution under vacuum for 2 h at room temperature, then dried the core to a constant weight at 105 °C. The contact angle of the dried core was tested by using a contact angle measuring instrument. Meanwhile, the surface wetting performance of fracturing fluid on the core was evaluated [46].

### 4.9. Natural Imbibition Experiment

Natural imbibition experiments were carried out through cores with numerous capillary throats. Firstly, the cores were dried at 105 °C for 4 h and immersed in the superhydrophobic gel fracturing fluid for 12 h. The cores were then dried to constant weight. The cores were mounted on an electronic balance to ensure that the cut surface of the core was tangent to the upper surface of the test solution. The mass of the test solution remaining in the beaker was weighed to obtain a time-dependent variation of the core’s natural sorption [47].

### 4.10. Formation Flowback Rate

**Figure 17:**
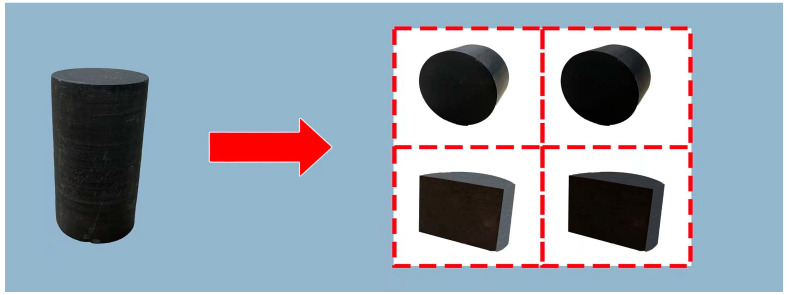
Schematic diagram of rock core being cut.

The target block cores were cut into two sets of cores of equal volume and mass. They are noted as No. 1, No. 2, No. 3, and No. 4, as shown in Figure 17. There was one group of No. 1 and No. 2 and one group of No. 3 and No. 4, then a control experiment was conducted. First, the four cores were dried and weighed to record, and the basic data of its core is shown in Table 4. Cores 1 and 3 were immersed in a mixture of superhydrophobic gel fracturing fluid and 3% KCl at 60 °C and 4.5 MPa for 7 h. Cores 2 and 4 were immersed in a mixture of ordinary polyacrylamide fracturing fluid and 3% KCl at 60 °C and 4.5 MPa for 7 h. Then, the 4 cores were immersed in equal volumes of tap water at 60 °C and 4.5 MPa for 5 h (the cores were suspended in the middle of the tap water with a strainer and did not touch the walls and bottom of the cup). The mass of the cores was recorded every 30 min until the mass of the 4 cores was constant [48].

### 4.11. Core Damage Performance Experiment

**Figure 18:**
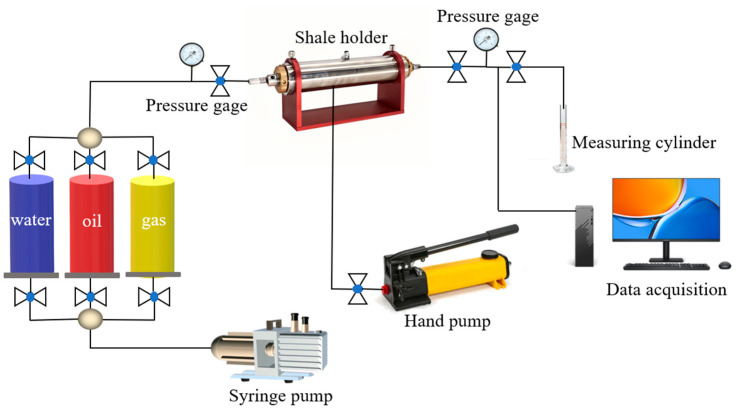
Schematic diagram of core displacement experiment.

In the process of oil and gas development, the permeability recovery value depends on the influence of multiple factors. A higher permeability recovery value means that oil and gas can flow out of the reservoir better. Generally, when the permeability recovery value is higher than 70%, it is considered to have a good reservoir protection effect. Therefore, according to the oil and gas industry standard SY/T 5107-2016 [49]. The damage performance of superhydrophobic gel fracturing fluid on the permeability of the core of the target block was evaluated. The gas flooding experimental setup is shown in Figure 18.

## Figures and Tables

**Table 1:** Effect of fracturing fluid on core permeability.

Number	Initial Permeability/mD	Permeability After Pollution/mD	Permeability Recovery Value/%	Average Permeability Recovery Value/%
1#	7.6068	6.4821	85.2	86.0
2#	10.2030	8.7590	85.8	
3#	12.1380	10.5607	87.0	

**Table 2:** Whole rock analysis of core.

Well Depth/m	Mineral Content/%							
	Quartz	Potassium Feldspar	Plagioclase	Calcite	Dolomite	Analcime	Barite	Clay Mineral
3880–3920	14.3	/	1.6	3.2	9.6	/	33.4	37.9

**Table 3:** Clay analysis of core.

Well Depth/m	Relative Content of Clay Minerals/%						Mixed Layer Ratio	
	S	I/S	It	Kao	C	C/S	I/S	C/S
3880–3920	/	36	31	16	17	/	5	/

**Table 4:** Basic data of processed core samples.

Core Number	Dry Weight/g	Surface Area/cm^2^	Volume/cm^3^
1	13.626	19.7127	6.1551
2	14.316	19.5188	6.0259
3	13.942	21.3260	6.2448
4	13.525	21.5859	6.3887

## Data Availability

The data presented in this study are openly available in article.

## Abbreviations

The following abbreviations are used in this manuscript:

Symbol	Description
SN-DR	Superhydrophobic nano-viscous drag reducer
FT-IR	Fourier transform infrared spectroscopy
^1^H NMR	^1^H Nuclear magnetic resonance spectroscopy
TGA	Thermogravimetric analysis
SEM	Scanning electron microscope
EDS	Energy Dispersive X-ray Spectroscopy
G′	Storage modulus (Unit: Pa)
G″	Loss modulus (Unit: Pa)
